# Peer review of "Improved Protocol for Efficient Agrobacterium-Mediated Transient Gene Expression in Medicago sativa L."

_plants, 2024, doi:10.3390/plants13212992_

Round 1

Reviewer 1 Report

Comments and Suggestions for Authors

This manuscript reports a thorough examination of variables for efficiency of transient transformation of alfalfa. My primary concern is that it is unclear if the GUS straining was due to expression from Agrobacterium rather than from transformed plant cells. Was a GUS gene with an intron used? If not, then the authors should include a disclaimer that they could not distinguish if expression of the marker gene was from Agrobacterium or from plant cells. The pattern of “dark to light blue within single or multiple cells” (Lines 172-173) suggests that some expression was from Agrobacterium. Another concern is that there is no verification that plant cell transformation occurred such as PCR showing chromosomal integration or transgenic plant regeneration. The paper would have greater impact if the authors could show that their improved protocol is applicable to other alfalfa germplasm that is recalcitrant to transformation. Regen-SY is reported to be easily transformable, while other germplasm is less transformable. It is also troubling that the protocol used with the GFP construct resulted in “severe tissue necrosis” (line 395). Was this construct in the same Agrobacterium strain and the improved protocol used? Why did this negative outcome occur? Such necrosis would likely reduce or eliminate regeneration. The tissue necrosis needs to be explained.

There are quite a few poorly explained figures. 

In figures 3 to 11 the Y axis is labeled % Transient GUS Expression and in the legend is described as GUS gene expression relative to the non-treated control. This is not logical. The untreated control would have 0% GUS expression so that each treated sample would be compared to 0% (or divided by 0). Instead, I think the authors are actually reporting the percentage of GUS positive explants. In the materials and methods, they define a GUS positive leaf as that with 25% or more of the tissue with blue spots (line 597) and indicate that they are measuring the percentage of GUS positive explants (line 630). The axis label and the figure legends should be revised to accurately report what was measured, % GUS positive explants. It is not clear what “standard error +/- 5” means. A standard error is a calculation from the mean. This needs to be corrected or explained. Each legend also indicates that there were significant differences but there is no notation of what treatments resulted in significantly different results. Parametric post hoc tests (t-test, Duncan's) were used on percentage data, which is not normally distributed. Nonparametric tests should be used instead. Also, it appears that leaflets were used in the experiments, not entire leaves (alfalfa is trifoliate).

Figure 3: In part A, was the leaflet entire or was a leaf piece used? In part C, excised is not correct. This means removed from the plant. Perhaps use sectioned instead.

Figure 4: The treatments need to be explained in the legend.

Figure 5: What tissue was used? Leaflet? Wounded? Age? Please add this information.

Figure 6: How were leaflets treated, what age was used?

Figure 7 to 11: Add that leaf explants were used in the legend. The figures need to stand independently from the text.

Much of the Results section contains material that is more appropriate in a Discussion. The Discussion section repeats some of this information. I suggest having a combined Results and Discussion section and deleting the Discussion section.

Line 531: The source of the explants is confusing. Were plants that were grown in GA7 boxes transplanted to soil and grown in a growth chamber? Or were sterile cuttings made and propagated in sterile culture? Was MS medium with sucrose used in the GA7 boxes? The term inoculated is confusing here (line 532). Do you mean the seedlings were moved to GA& boxes?

Line 569: What is the “prepared Agrobacterium”? Were cells pelleted and resuspended or was the culture in LB broth used for explant inoculation?

Comments on the Quality of English Language

I have noted where the text is confusing and inaccurate in my comments above.

Author Response

Thank you for your comments and spending time concerning our manuscript. The comments are very helpful for improving our manuscript. We tried to address all the comments very carefully and have made the correction accordingly. Revised sections are marked with different color (red) in the manuscript. Our responses are given below:

Reviewer 1:

Comment 1. This manuscript reports a thorough examination of variables for efficiency of transient transformation of alfalfa. My primary concern is that it is unclear if the GUS straining was due to expression from Agrobacterium rather than from transformed plant cells. Was a GUS gene with an intron used? If not, then the authors should include a disclaimer that they could not distinguish if expression of the marker gene was from Agrobacterium or from plant cells. The pattern of “dark to light blue within single or multiple cells” (Lines 172-173) suggests that some expression was from Agrobacterium. Another concern is that there is no verification that plant cell transformation occurred such as PCR showing chromosomal integration or transgenic plant regeneration. The paper would have greater impact if the authors could show that their improved protocol is applicable to other alfalfa germplasm that is recalcitrant to transformation. Regen-SY is reported to be easily transformable, while other germplasm is less transformable. It is also troubling that the protocol used with the GFP construct resulted in “severe tissue necrosis” (line 395). Was this construct in the same Agrobacterium strain and the improved protocol used? Why did this negative outcome occur? Such necrosis would likely reduce or eliminate regeneration. The tissue necrosis needs to be explained.

Response: We agree with this comment. Therefore, we have addressed all the information, Figure 1: Please see the lines 127-138 for Agrobacterium construction. Figure 14: the lines 437-453 of PCR analysis for the verification of transformed alfalfa plant cell. Please see the lines 408-416 for severe tissue necrosis.  We put our agro-infected explants on the hygromycin selection medium that causes the tissue necrosis and allow to grow only the transformed cells.

Comment 2. There are quite a few poorly explained figures. In figures 3 to 11 the Y axis is labeled % Transient GUS Expression and in the legend is described as GUS gene expression relative to the non-treated control. This is not logical. The untreated control would have 0% GUS expression so that each treated sample would be compared to 0% (or divided by 0). Instead, I think the authors are actually reporting the percentage of GUS positive explants. In the materials and methods, they define a GUS positive leaf as that with 25% or more of the tissue with blue spots (line 597) and indicate that they are measuring the percentage of GUS positive explants (line 630). The axis label and the figure legends should be revised to accurately report what was measured, % GUS positive explants. It is not clear what “standard error +/- 5” means. A standard error is a calculation from the mean. This needs to be corrected or explained. Each legend also indicates that there were significant differences but there is no notation of what treatments resulted in significantly different results. Parametric post hoc tests (t-test, Duncan's) were used on percentage data, which is not normally distributed. Nonparametric tests should be used instead. Also, it appears that leaflets were used in the experiments, not entire leaves (alfalfa is trifoliate).

Response: As the reviewer’s suggestion we have corrected the figures and explanation. Please see the revised manuscript figure 4-12.

Comment 3. Figure 3: In part A, was the leaflet entire or was a leaf piece used? In part C, excised is not correct. This means removed from the plant. Perhaps use sectioned instead.

Response: We agreed your suggestion; in figure 4A we used leaflet (one leaf) from trifoliate alfalfa. We have corrected Figure 4C, with the term ‘sectioned’.

Comment 4. Figure 4: The treatments need to be explained in the legend.

Response: Please see the figure 5 in the revised version. We already have explained the treatments in the lines 632-634 at the material and methods part.

Comment 5. Figure 5: What tissue was used? Leaflet? Wounded? Age? Please add this information.

 Response: According to our experiment, we discussed in the Figure 4a, 3-week-old fully expanded leaf were cut into 3 pieces and the sectioned leaf (figure 4c) were wounded with scalpel (Figure 5).

Comment 6. Figure 6: How were leaflets treated, what age was used?

Response: We used fully expanded leaves from 3-week-old alfalfa plants (figure 4B) that were carefully removed and cut into small segments of uniform size (≈2 mm). Leaf explants were placed on a callus induction medium using Gamborg's B5 basal (B5H) medium, supplemented with 3% maltose, 4.5 μM 2,4-dichlorophenoxyacetic acid (2,4-D), 0.9 μM kinetin, 6.65 g of glutamine, 0.8 g of serine, 0.004 g of adenine, and 0.08 g of L-glutathione. The cultures were incubated in the dark at 24 ± 2 ℃ for 3 weeks.  Please see the Lines 588-595 at the material and method section and Figure 7 at our revised version

 Comment 7. Figure 7 to 11: Add that leaf explants were used in the legend. The figures need to stand independently from the text.

Response: As for the reviewer suggestion, we have added the leaf explant were used in our experiment from figure 8-12.

Comment 8. Much of the Results section contains material that is more appropriate in a Discussion. The Discussion section repeats some of this information. I suggest having a combined Results and Discussion section and deleting the Discussion section.

Response: As suggested by the three other reviewers, we decided to keep the format in the result and discussion separately.

Comment 9. Line 531: The source of the explants is confusing. Were plants that were grown in GA7 boxes transplanted to soil and grown in a growth chamber? Or were sterile cuttings made and propagated in sterile culture? Was MS medium with sucrose used in the GA7 boxes? The term inoculated is confusing here (line 532). Do you mean the seedlings were moved to GA& boxes?

Response: Thank you for pointing this out. The alfalfa plants were grown and subcultured in the sterile MS basal medium with sucrose in the GA7 boxes. The germinated seedlings were moved to GA7 boxes.

 Comment 10. Line 569: What is the “prepared Agrobacterium”? Were cells pelleted and resuspended or was the culture in LB broth used for explant inoculation?

Response: As the reviewer suggested we have used the term “overnight grown” instead of the “prepared agrobacterium”. Please see the line 620.

Once again, we wish to thank the review team and editors for their time reviewing and providing input for our manuscript. We feel our manuscript significantly improved following both rounds of reviews and we are grateful for all of their time, effort, and advice on how to improve our work.

Reviewer 2 Report

Comments and Suggestions for Authors

The MS entitled "Improved Protocol for Efficient Agrobacterium-mediated Transformation in Medicago sativa L" studies the effect of various parameters such as explant type, age, preincubation period, cocultivation time, Agrobacterium concentration, use of additives such as silver nitrate, Acetosyringone on T-DNA transfer in Medicago sativa and used GUS staining and GFP visualization as methods to verify successful T-DNA transfer.

The scientific design of the MS is fine but the presentation of results has serious flaws. I have included my comments and highlighted the issues directly in the PDF file which is attached here.

In the results section, the authors made several claims which are not supported by any data. I suggest removing them from the results section and including them in the discussion section with appropriate references.

The authors have mentioned using controls in their experiments but did not include controls for GUS staining images in Fig. 2.

The authors have standardized various parameters for efficient T-DNA delivery but they have not performed any experiment to include all the standardized conditions in a single experiment to show if these parameters have any additive effect on T-DNA delivery.

The authors are also suggested to include an experiment to test the ability of different Agrobacterium strains to deliver T-DNA in alfalfa.

Author Response

Thank

Thank you for your comments and spending time concerning our manuscript. The comments are very helpful for improving our manuscript. We tried to address all the comments very carefully and have made the correction accordingly. Revised sections are marked with different color (red) in the manuscript. Our responses are given below:

Comment 1: The MS entitled "Improved Protocol for Efficient Agrobacterium-mediated Transformation in Medicago sativa L" studies the effect of various parameters such as explant type, age, preincubation period, cocultivation time, Agrobacterium concentration, use of additives such as silver nitrate, Acetosyringone on T-DNA transfer in Medicago sativa and used GUS staining and GFP visualization as methods to verify successful T-DNA transfer. The scientific design of the MS is fine but the presentation of results has serious flaws. I have included my comments and highlighted the issues directly in the PDF file which is attached here. In the results section, the authors made several claims which are not supported by any data. I suggest removing them from the results section and including them in the discussion section with appropriate references.

Response: Thank you for your suggestion. We have addressed the comments in the results and discussion sections. Please see the revised version of our manuscript.

Comment 2: The authors have mentioned using controls in their experiments but did not include controls for GUS staining images in Fig. 2.

Response: We added the control or ‘0’ for each parameters in the figure. Please see the figure 4-12 in the revised version according to your suggestion.

Comment 3: The authors have standardized various parameters for efficient T-DNA delivery but they have not performed any experiment to include all the standardized conditions in a single experiment to show if these parameters have any additive effect on T-DNA delivery.

Response: Thank you for your valuable comments. We added the PCR result for the confirmation with all the standardized conditions in a single experiment.

Comment 4: The authors are also suggested to include an experiment to test the ability of different Agrobacterium strains to deliver T-DNA in alfalfa.

Response: Thank you very much for your suggestions. We chose the pCAMBIA1304 plasmid as it is already built up with two reporter genes, GUS and GFP and hygromycin as a selectable marker.

Once again, we wish to thank the review team and editors for their time reviewing and providing input for our manuscript. We feel our manuscript significantly improved following both rounds of reviews and we are grateful for all of their time, effort, and advice on how to improve our work.

Reviewer 3 Report

Comments and Suggestions for Authors

This manuscript describes a protocol for achieving efficient transient gene expression in alfalfa through genetic transformation with the Agrobacterium. The genetic engineering offers a faster route for trait modification and improvement. The manuscript is clearly written and the figures effectively illustrate the results.

Comments:

1. The TITLE: This article mainly focus on a protocol for achieving efficient transient gene expression in alfalfa through genetic transformation with the Agrobacterium, but the title does not show the theme of transient gene expression.

2. This article incorporates dual reporter genes, GUS and GFP. Are the results of these two reporter genes consistent? Are the expression levels of GFP and GUS consistent?

3. There is no bar in Fig1, Fig2, and Fig12.

4. From Fig3 to Fig11, the main title (Optimization of transient GUS gene expression in alfalfa) are the same. Can that change AND simplify?

5. In Lane309, Acetatosyringone should be abbreviated as AS.

6. In Lane686, wel should be well.

7. In Lane687, at the do not use superscript fonts. 

Author Response

Thank you for your comments and spending time concerning our manuscript. The comments are very helpful for improving our manuscript. We tried to address all the comments very carefully and have made the correction accordingly. Revised sections are marked with different color (red) in the manuscript. Our responses are given below:

Comment 1. The TITLE: This article mainly focus on ‘a protocol for achieving efficient transient gene expression in alfalfa through genetic transformation with the Agrobacterium’, but the title does not show the theme of ‘transient’ gene expression.

Response: Thank you for your suggestion. According to the reviewer advice, we already modified the title of our manuscript.

Comment 2. This article incorporates dual reporter genes, GUS and GFP. Are the results of these two reporter genes consistent? Are the expression levels of GFP and GUS consistent?

Response: The expression levels of the two reporter genes, GUS and GFP, were consistent in explant used for transient gene expression. Please see the lines 414-416 in the results section.

Comment 3. There is no bar in Fig1, Fig2, and Fig12.

Response: Thank you for your advice. We improved our figure 2, 3, and 13 in our revised version and added bar in all the figures.

Comment 4. From Fig3 to Fig11, the main title (Optimization of transient GUS gene expression in alfalfa) are the same. Can that change AND simplify?

Response: According to the reviewer suggestions, we have modified and tried to simplify the title of all the figures. Please see the figures 4-12 in the results section.

Comment 5. In Lane309, Acetosyringone should be abbreviated as AS.

Response: Thank you for the comment. We replaced AC with AS. Please see the lines 325-345 in the results section and lines 511-524 in the discussion section.

Comment 6. In Lane686, ‘wel’ should be ‘well’.

Response: We have changed wel to well. Please see the line 752.

Comment 7. In Lane687, ‘at the’ do not use superscript fonts. 

Response: We have changed. Please see the line 754.

Once again, we wish to thank the review team and editors for their time reviewing and providing input for our manuscript. We feel our manuscript significantly improved following both rounds of reviews and we are grateful for all of their time, effort, and advice on how to improve our work.

Reviewer 4 Report

Comments and Suggestions for Authors

Manuscript entitledImproved Protocol for Efficient Agrobacterium-mediated Transformation in Medicago sativa L.. This manuscript described a protocol for achieving efficient transient gene expression in alfalfa through genetic transformation with the Agrobacterium tumefaciens pCAMBIA1304 vector. However, several points need to be addressed before accepted.

1. This research optimize critical parameters that positively influence Agrobacterium-mediated transformation efficiency during the infection and co-cultivation stages. However, orthogonal experimental or response surface design was absent to analyze the optimal parameters during Agrobacterium-mediated transformation.

2.  Agrobacterium-mediated transformation in Medicago sativa L. had been previously reported, therefore, the innovation of this study should be added in the Introduction section.

3. Line 547-548. What is the source of pCAMBIA1304 plasmid expressing both 547 GUS and GFP gene fusion? Is the pCAMBIA1304 plasmid constructed in this study or previously obtained?

4. Line 547-548. The steps of Agrobacterium-mediated transformation in Medicago sativa L. should be detailed described.

5. Line 570. The steps of pCAMBIA1304 transformed in Agrobacteriumshould be detailed described. 

Comments on the Quality of English Language

Minor editing of English language required.

Author Response

Thank you for your comments and spending time concerning our manuscript. The comments are very helpful for improving our manuscript. We tried to address all the comments very carefully and have made the correction accordingly. Revised sections are marked with different color (red) in the manuscript. Our responses are given below:

Manuscript entitled “Improved Protocol for Efficient Agrobacterium-mediated Transformation in Medicago sativa L.”. This manuscript described a protocol for achieving efficient transient gene expression in alfalfa through genetic transformation with the Agrobacterium tumefaciens pCAMBIA1304 vector. However, several points need to be addressed before accepted.

Comment 1. This research optimize critical parameters that positively influence Agrobacterium-mediated transformation efficiency during the infection and co-cultivation stages. However, orthogonal experimental or response surface design was absent to analyze the optimal parameters during Agrobacterium-mediated transformation.

Response: Thank you for your suggestion. We added the control or ‘0’ for each parameters in the figure. Please see the figure 4-12 in the revised version.

Comment 2.  Agrobacterium-mediated transformation in Medicago sativa L. had been previously reported, therefore, the innovation of this study should be added in the Introduction section.

Response: We agreed with your comments and added the rational of our study in the introduction section. Please see the lines 113-126.

Comment 3. Line 547-548. What is the source of pCAMBIA1304 plasmid expressing both 547 GUS and GFP gene fusion? Is the pCAMBIA1304 plasmid constructed in this study or previously obtained?

Response: As suggested by the reviewers, we have added the map of pCAMBIA1304 plasmid expressing both GUS and GFP gene fusion. We have put the legends with the source of the map that explains we have previously obtained the plasmid. Please see the lines 127-138.

Comment 4. Line 547-548. The steps of Agrobacterium-mediated transformation in Medicago sativa L. should be detailed described.

Response: According to the reviewer’s suggestion, we have included the detail description in the Figure 15 at our revised version in the manuscript that describes the step by step protocol in our experiment.

Comment 5. Line 570. The steps of pCAMBIA1304 transformed in Agrobacterium should be detailed described.

Response: As suggested by the reviewer, we are happy to provide the source of agrobacterium, which was obtained from Dr. Amit Dhingra. We also acknowledged him for providing us with the pCAMBIA1304 vector in the acknowledgment section.

Once again, we wish to thank the review team and editors for their time reviewing and providing input for our manuscript. We feel our manuscript significantly improved following both rounds of reviews and we are grateful for all of their time, effort, and advice on how to improve our work.

Round 2

Reviewer 1 Report

Comments and Suggestions for Authors

The authors have mostly responded to the comments from my previous review. Below are specific recommendations.

Line 125: Not clear why the authors are influencing transient expression rather than stable transformation. Perhaps end the sentence at alfalfa. (advance gene-editing techniques in alfalfa to influence transient gene expression.)

Line 130: Figure 1 is a circular map although the legend says linear. Suggest this edit: The plasmid vector pCAMBIA1304 used for the transformation of various alfalfa tissues.

Line 582: This was not changed (were inoculated in the magenta GA7 boxes) from the previous version. Plants were not inoculated they were transferred to GA7 boxes. Please add what medium was used in the GA7 boxes.

Line 638: It is not clear if the CaCl2 and silver nitrate was in the culture medium or the inoculation solution. Please add this information.

Line 678: The PCR assay adds little new information. It is not proof that the alfalfa cells were transformed. The same fragment would be amplified from Agrobacterium with the plasmid. The antibiotics used after co-cultivation inhibit bacterial growth but do not eliminate the bacteria.

Comments on the Quality of English Language

Moderate editing and formatting for consistency is needed.

Author Response

Reviewer 1

Line 125: Not clear why the authors are influencing transient expression rather than stable transformation. Perhaps end the sentence at alfalfa. (advance gene-editing techniques in alfalfa to influence transient gene expression.)

Answer: Our experimental findings suggest that the transient gene expression system was designed to improve the production of transgenic plants and, in the long term, advance gene-editing techniques in alfalfa. We have also made corrections to lines 124-125.

 Line 130: Figure 1 is a circular map although the legend says linear. Suggest this edit: The plasmid vector pCAMBIA1304 used for the transformation of various alfalfa tissues.

Answer: Thank you for your comments. We corrected the legend in Figure1.

Line 582: This was not changed (were inoculated in the magenta GA7 boxes) from the previous version. Plants were not inoculated they were transferred to GA7 boxes. Please add what medium was used in the GA7 boxes.

Answer: We added the medium composition. Please see the materials and method section and lines 578-579. “The Petri dishes were kept in the dark for 3 weeks at 24°C. After 3 weeks, nine germinated seeds per petri dish were transferred in the magenta GA7 boxes containing MS basal medium with 3% sucrose and 0.8% w/v agar and incubated at a temperature of 24°C for 7 days in a growth chamber supplied with a 16/8 h photoperiod using cool, white, fluorescent light (75 lmol s-1 m-2).”

Line 638: It is not clear if the CaCl2 and silver nitrate was in the culture medium or the inoculation solution. Please add this information.

 Answer: We agreed with your suggestion. We added the CaCl2 and silver nitrate in the culture medium. We included this information in our revised version lines 634-635.

Line 678: The PCR assay adds little new information. It is not proof that the alfalfa cells were transformed. The same fragment would be amplified from Agrobacterium with the plasmid. The antibiotics used after co-cultivation inhibit bacterial growth but do not eliminate the bacteria.

Answer: Please see Materials and method section line 685-689 “Before the PCR analysis, selected transgenic tissue segments were cultured in the LB medium for 36-48 hours on 200 rpm in the shaker. By visual observation, no precipitation or change in color was observed in the LB medium which indicates the absence of Agrobacterium in the transgenic tissue. To double confirm, we checked the O.D. of the transgenic tissue growing liquid medium and control (LB medium) and found the same results.’’

Also, please see 2.11 sectionMolecular Analysis on Transgenic Tissue” in the Results line 425-439 The selected explants were transferred to Gamborg's B5 basal (B5H) medium, supplemented with 3% maltose, 4.5 μM 2,4-dichlorophenoxyacetic acid (2,4-D), 0.9 μM kinetin, 6.65 g/L glutamine, 0.8 g/L serine, 0.004 g/L adenine, 0.08 g/L L-glutathione, 400 mg/L cefotaxime, and 12.5 mg/L hygromycin. After 3 weeks, callus formation was initiated, followed by somatic embryo development. The putative transgenic plants were regenerated from the matured embryos. To confirm the presence of stable GUS gene expression in alfalfa putative transformant explants (leaf, stem, petiole, root, and 3-week-old leaf-derived callus), polymerase chain reaction (PCR) amplification was performed on their genomic DNA. Genomic DNAs of alfalfa were extracted from both putative transformed and non-transformed explants. Using GUS-specific primers, a PCR product of the expected 493 bp fragment was detected in all transformed explants (Lanes 2-6), which tested positive for GUS in the GUS assay, while no amplification was observed in non-transformed explants (Lanes 7-11) (Figure 14). The detection of the 493 bp GUS fragment by PCR aligned with reference genes, confirming the presence of the GUS gene through transient GUS gene expression and reducing the likelihood of false positives.

Reviewer 2 Report

Comments and Suggestions for Authors

The authors have made several changes in the MS, which certainly improved its quality, but several issues remain that need to be addressed.

My comments on the revised version of this MS are attached. 

Reviewer 4 Report

Comments and Suggestions for Authors

Orthogonal experimental or response surface design was necessary to analyze the optimal parameters combination. This study only uses single factor test to optimize the condition of Agrobacterium-mediated transformation. Therefore, the author needs to conduct orthogonal experimental or response surface design for optimize the condition of Agrobacterium-mediated transformation, or to interpret why not use orthogonal experimental or response surface design in this study in Discussion section.

Author Response

Reviewer 3

Orthogonal experimental or response surface design was necessary to analyze the optimal parameters combination. This study only uses single factor test to optimize the condition of Agrobacterium-mediated transformation. Therefore, the author needs to conduct orthogonal experimental or response surface design for optimize the condition of Agrobacterium-mediated transformation, or to interpret why not use orthogonal experimental or response surface design in this study in Discussion section.

Answer: Our research focuses on identifying the optimal parameters required to enhance transient gene expression by using the appropriate factors. In our view, testing with an orthogonal design that includes all possible interactions is time-consuming, and accounting for additional, non-essential factors is impractical for our study. Our goal was to produce the transgenic alfalfa plants by improving the transient gene expression in alfalfa that provides the parameters for efficient and reproducible stable transformation.

Round 3

Reviewer 2 Report

Comments and Suggestions for Authors

The authors have made required changes in the MS after second round of review and I am satisfied with the changes.